# Top-Down N-Doped Carbon Quantum Dots for Multiple Purposes: Heavy Metal Detection and Intracellular Fluorescence

**DOI:** 10.3390/nano11092249

**Published:** 2021-08-31

**Authors:** Francesca Limosani, Elvira Maria Bauer, Daniele Cecchetti, Stefano Biagioni, Viviana Orlando, Roberto Pizzoferrato, Paolo Prosposito, Marilena Carbone

**Affiliations:** 1 Department of Industrial Engineering, University of Rome Tor Vergata, Viale del Politecnico 1, 00133 Rome, Italy; francesca.limosani@uniroma2.it (F.L.); pizzoferrato@uniroma2.it (R.P.); Paolo.Prosposito@uniroma2.it (P.P.); 2 Department of Chemical Science and Technologies, University of Rome Tor Vergata, Via della Ricerca Scientifica 1, 00133 Rome, Italy; Daniele.Cecchetti@uniroma2.it; 3 Institute of Structure of Matter (CNR-ISM), Italian National Research Council, Via Salaria km 29.3, 00015 Monterotondo, RM, Italy; elvira.bauer@ism.cnr.it; 4 Department of Biology and Biotechnology “Charles Darwin”, Sapienza University of Rome, P.le A. Moro, 00185 Rome, Italy; stefano.biagioni@uniroma1.it (S.B.); viviana.orlando@uniroma1.it (V.O.)

**Keywords:** fullerene, carbon quantum dots, heavy metals, photoluminescence, bioimaging, quenching, spectroscopy

## Abstract

In the present study, we successfully synthesized N-doped carbon quantum dots (N-CQDs) using a top-down approach, i.e., hydroxyl radical opening of fullerene with hydrogen peroxide, in basic ambient using ammonia for two different reaction times. The ensuing characterization via dynamic light scattering, SEM, and IR spectroscopy revealed a size control that was dependent on the reaction time, as well as a more pronounced -NH_2_ functionalization. The N-CQDs were probed for metal ion detection in aqueous solutions and during bioimaging and displayed a Cr^3+^ and Cu^2+^ selectivity shift at a higher degree of -NH_2_ functionalization, as well as HEK-293 cell nuclei marking.

## 1. Introduction

Carbon-based nanomaterials, such as zero-dimensional fullerenes, 1D carbon nanotubes, and 2D graphene, have received considerable attention as useful materials for various applications in electronics, optoelectronics, photovoltaics, and sensing [1,2,3]. Carbon quantum dots (CQDs) not only inherit the excellent optical properties of traditional semiconductor quantum dots [4,5] but also compensate for the deficiencies of the traditional materials in terms of biocompatibility, cytotoxicity, and biohazards [6,7]. Other relevant aspects of these nanomaterials are their excellent solubility in an aqueous environment, chemical stability, photobleaching resistance, large-scale preparation, and ease of surface functionalization [8]. Due to quantum confinement effects and localized surface states, CQDs exhibit a variable photoluminescence emission [9,10]. The subsequent interaction of ions or molecules can modify the localized surface states, thus causing a photoluminescence quenching or enhancement effect. As a consequence, these materials are promising candidates for replacing the metal-based quantum dots in various applications, such as bioimaging [11,12,13], biosensing [14,15], drug delivery [16,17], adjuvant selection in vaccines [18], and photocatalysis [19]. The working mechanism in sensing and imaging applications is related to the presence of functional groups, which can coordinate the metals being detected, thus affecting the optical properties. The coordinating role is often played by oxygenated functional groups, such hydroxyls and carboxylates, that are attached to CQDs, but additional N-doping is sought for shifting selectivity parameters or spectral features [20].

The efficacy of the CQDs for detection purposes needs to meet application requirements, which privilege facile syntheses without further treatments prior to their use, in order to achieve a practical ready-to-use tool. Important progress in the synthesis methods of these materials was achieved, adopting either bottom-up or top-down approaches, depending on starting materials, target size, and target ion detection. In the case of bottom-up approaches, the starting point is a mix of small organic molecules that undergo pyrolysis. Through polymerization, the carbonization of the precursors can be achieved by means of microwave, ultrasonication, hydrothermal, or solvothermal treatments. Citric acid condensation or pyrolysis is the progenitor of a set of bottom-up syntheses [21], which was used in a large number of applications [22,23]. In a typical synthesis, CQDs are synthesized using citric acid pyrolysis at 180–200 °C, followed by water addition upon color changing of the solution to pale yellow/orange, centrifugation, and dialysis (a lengthy, yield-affecting procedure). Bottom-up strategies were further extended to the condensation of saccharides [24], biopolymers [25], ascorbic acid [26], and humic acid [27], as well as different plant species, including fruits and vegetables [28], and waste materials, such as waste-paper and frying oil [29]. In terms of ion detection, pyrolysis-derived CQDs are usually sensitive to Fe^3+^ or Fe^2+^ only [30]. N-doping in the bottom-up synthesis of CQDs is achieved using a hydrothermal procedure after mixing a reagent acting as a C-donor and one acting as a N-donor. Such mixtures include CCl_4_/1,2-ethylenediamine, CCl_4_/1,3-propanediamine, CCl_4_/1,4-butanediamine [31], citric acid/glycine [32], folic acid/ethylene glycol [33], and a mixture of seaweed-derived κ-carrageenan/lemon juice/belzalkonium chloride [34] and shift the selectivity to multiple ions [31] or different ions [33]. Although in some cases, the bottom-up synthesized N-doped CQDs (N-CQDs) appear to be effective, reagents such as CCl_4_ are toxic or even carcinogenic and consequently banned from lab use in some countries. In other cases, the source of nitrogen is part of the biomass, such as in rice residues, whose pyrolysis generated the N-CQDs [35], or Tulsi leaves, which yielded N-CQDs by hydrothermal treatment after fine grinding [36]. The top-down synthesis of CQDs is derived from the separation of large carbon precursors, which are usually prepared from carbonic materials, including nanotubes, graphene, carbon black, and fullerene. These carbonic materials with an sp^2^ carbon structure are abundant but have an infinite Bohr diameter and lack an effective bandgap to produce luminesce on excitation [37]. Thus, breaking down these large carbon sources into nanoscale particles is an essential step to endow them with photoluminescence (PL) through quantum confinement effects [38]. This can usually be achieved via physical and chemical methods, such as laser ablation and electrochemistry, as well as redox reactions, which are variants of the Hummer method and employ sulfonitric attacks [39]. The redox top-down synthesis, especially when applied to fullerene, allows for size control due to the unfolding of regularly sized units, such as Buckminster buckyballs. The opening or unfolding procedure leaves oxygen-containing functional groups, such as hydroxyls or carboxylic acids on the fullerene halves. However, N-doping ensuing unfolding is not a common procedure.

In the present paper, we present a simplified and low-cost top-down synthesis for obtaining N-doped CQDs, with a size control that depends on the initial material (fullerene) and can be further refined through the reaction time. The synthesized N-CQDs were subsequently used as fluorescent probes for metal ion detection sensing and imaging of cell nuclei. In defining the synthetic strategy, we privileged easiness, i.e., we proceeded with a one-step synthesis, that did not require a hydrothermal treatment, and avoided the dialysis procedure, which can be costly and lengthy, using fullerene as the starting material for better size control. Since the opening requires an active breaking action, we used a mixture of hydrogen peroxide (H_2_O_2_) and ammonia (NH_4_OH), which exerts their opening action via oxidation, and provides both of the required oxygenated and nitrogenated functional groups. The whole procedure was followed by sheer centrifugation. This simple procedure was carried out for different lengths of time to determine possible differences. The N-CQDs were, then, characterized using dynamic light scattering (DLS), scanning electron microscope (SEM) imaging, and infrared spectroscopy. The efficacy of the N-CQDs was probed in the application as fluorescent tools for metal ion detection in water and cell imaging. We found that longer reaction times for the N-CQDs’ preparation favored the introduction of a larger amount of -NH_2_. The concomitant effects were a shift in selectivity toward Cu^2+^ and Cr^3+^ metal detection and more efficient imaging of cell nuclei. 

## 2. Materials and Methods

### 2.1. Materials and Equipment

Fullerene C60 flakes and hydrogen peroxide solution (30%) were purchased from Sigma-Aldrich (St. Louis, MO, USA). Ammonium hydroxide solution (28%) was ordered from VWR Chemicals (VWR International, Radnor, PA, USA). The water used in all the experiments was doubly distilled and purified using a Milli-Q system (Millipore, Milford, MA, USA). All metal salts (Cd(NO_3_)_2_·5H_2_O, Cu(NO_3_)_2_, AgNO_3_, CrCl_3_·6H_2_O, SnCl_2_·6H_2_O, ZnCl_2_, AlCl_3_) were purchased from Merck KGaA (Darmstadt, Germany). The metal salts solutions were prepared at the concentration of 10,000 µM. Infrared spectra were taken with a Shimadzu Prestige-21 FT-IR instrument (Shimadzu Corp., Nakagyo-ku, Kyoto, Japan), which was equipped with an attenuated total reflectance (ATR) diamond crystal (Specac Golden Gate, Specac Ltd., Orpington, Kent, UK), in the range 400–4000 cm^−1^, with a resolution of 4 cm^−1^. A layer of N-CQDs was deposited on a clean Al foil using drop-casting and the deposits were dried in air before measurements were taken. SEM images were taken with a Zeiss Auriga Field Emission–Scanning Electron Microscope instrument (Carl Zeiss Microscopy, Cambridge, UK) operating at 7 kV on N-CQDs diluted 1:100 with deionized water and deposited on a clean Si wafer surface. Measurements were taken upon complete solvent evaporation. The size distribution was measured using a Nano ZS90 (Malvern, UK) at room temperature using disposable plastic cuvettes. Fluorescence measurements were performed using a laboratory setup for photoluminescence, which was equipped with a 200 W Hg (Xe) continuous discharge lamp (Oriel Corp., Stratford, CT, USA) with an excitation 25 cm monochromator (Photon Technology International, Inc., Birmingham, NJ, USA) and an emission 25 cm monochromator (Cornerstone 260, Oriel Instruments, Stratford, CT, USA). The spectral response of the setup was calibrated over the wavelength range of interest using reference fluorophore solutions of quinine sulfate and a certified spectral fluorescence standard kit (Sigma-Aldrich) [40]. The samples were investigated in rectangular fused silica cuvettes with an optical length of 10 mm. The PL signal was collected at the conventional 90° geometry by quartz lenses with appropriate rejection filters. All the emission spectra were generally recorded using the excitation wavelength of λ_exc_ = 360 nm with a spectral bandwidth of approximately 3 nm. UV-Vis absorption spectra were recorded using a Cary 50 spectrophotometer (Varian Inc., Palo Alto, CA, USA). All the PL and UV-Vis spectra were elaborated with the Origin Pro program. The PL spectra were deconvolved using Gaussian functions to determine the peak position and the higher full width at half maximum (FWHM). Human embryonic kidney 293 (HEK-293) cells were cultured in Dulbecco’s Modified Eagle Medium (DMEM) supplemented with 2 mM glutamine and 10% fetal bovine serum (FBS) maintained in a humidified incubator at 10% CO_2_ and periodically tested to ensure the absence of mycoplasma contamination. Afterward, the cells were incubated with 2.2 mM N-CQDs-40 min or N-CQDs-15 h in DMEM for 30 min, 1 h, or 2 h. Nuclei of the cells were labeled with DAPI (4′,6-diamidin-2-fenilindolo) fixed in 4% paraformaldehyde for 15 min and then washed three times with PBS. Images were taken with an ApoTome System (Zeiss) that was connected to an AxioObserver Z1 inverted microscope (Carl Zeiss Microscopy GmbH, Jena, Germany). 

### 2.2. Synthesis of N-CQD-Based Nanomaterials 

**Figure 1:**
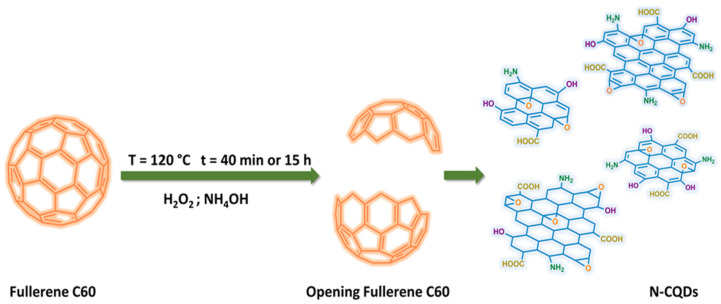
Preparation of fluorescent N-CQDs through hydroxyl radical-induced decomposition of fullerene C60.

The synthesis of N-CQD-based nanomaterials was performed using a hydroxyl-radical related method to open and cut down fullerene into N-CQDs. Ariga and co-workers reported the decomposition of H_2_O_2_ into hydroxyl radicals at a high NaOH concentration [41,42]. In order to increase the optical properties and electrocatalytic activities of the CQDs, heteroatom doping was evaluated to improve the properties and enhance the selectivity of these materials toward the detection of specific heavy metal ions. For this reason, NH_4_OH was used instead of NaOH during the synthetic process. In a three-neck round-bottom flask, 20 mL of fullerene that was dispersed in ultrapure water (5 mg/mL) was sonicated for 1 h 30 min. Afterward, N-CQDs were prepared by loading 4 mL of NH_4_OH (28%) to the fullerene dispersion and slowly adding 16 mL of H_2_O_2_ (30%). Subsequently, the resulting solution was heated in an oil bath at 120 °C for 40 min or for 15 h to produce N-CQDs. The final products were centrifuged at 4500 rpm for 10 min, followed by centrifugation at 15,000 rpm for 15 min. The precipitate was then removed and the supernatant of each solution was used for the subsequent analyses without any further treatment. The achieved samples were named N-CQDs-40 min and N-CQDs-15 h. The reaction scheme is shown in Figure 1.

## 3. Results

The synthesized samples were characterized with different techniques. In particular, the average size of the quantum dots was estimated using DLS and SEM imaging. The presence of functional groups on the surface of the quantum dots was assessed using infrared spectroscopy. Subsequently, the optical and fluorescence properties and metal detections were determined using absorption and fluorescence spectroscopies and the bioimaging was achieved using fluorescence microscopy.

### 3.1. Characterization of the N-CQDs-Based Nanomaterials

**Figure 2:**
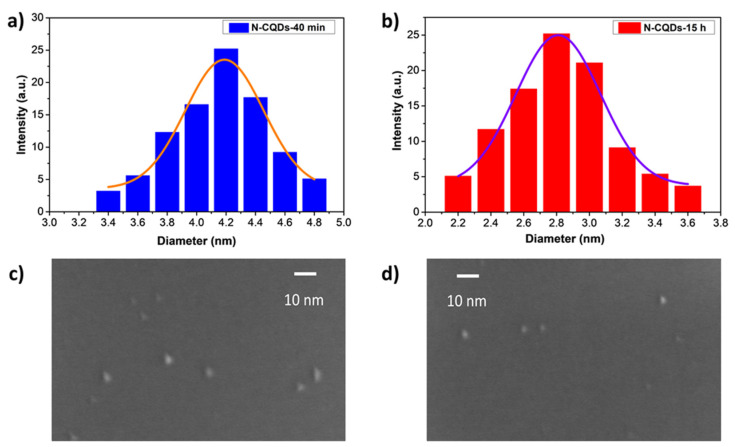
Particle size distribution measured using DLS: (**a**) N-CQDs-40 min and (**b**) N-CQDs-15 h; SEM images of (**c**) N-CQDs-40 min and (**d**) N-CQDs-15 h.

The average size of the N-CQDs was estimated using DLS. The size distribution is reported in Figure 2a,b for the two samples. It was 4.19 ± 0.02 nm for the 40 min preparation, decreasing to 2.81 ± 0.02 nm for the 15 h preparation, with a size distribution range of ±0.8 nm in both cases. This indicated the efficacy of the hydroxyl-radical-opening procedure, as well as the erosion of the quantum dots’ edges as an effect of the reaction time due to the continuous action of the reaction mixture. 

SEM images are reported in Figure 2c,d for diluted samples of N-CQDs-40 min and N-CQDs-15 h, respectively, and indicate the presence of CQDs in a size range that was in line with the DLS measurements. The shapes and boundaries, though difficult to estimate at these large magnifications, were irregular.

The introduction of functional groups on the unfolded moieties was evaluated using ATR-FT-IR spectroscopy, especially for determining the simultaneous presence of oxygen and nitrogen derivatives. ATR-FT-IR spectra of the QDs dispersions were registered after drop-casting the aqueous solutions on clean aluminum foils and subsequently drying the deposits in air. 

**Figure 3:**
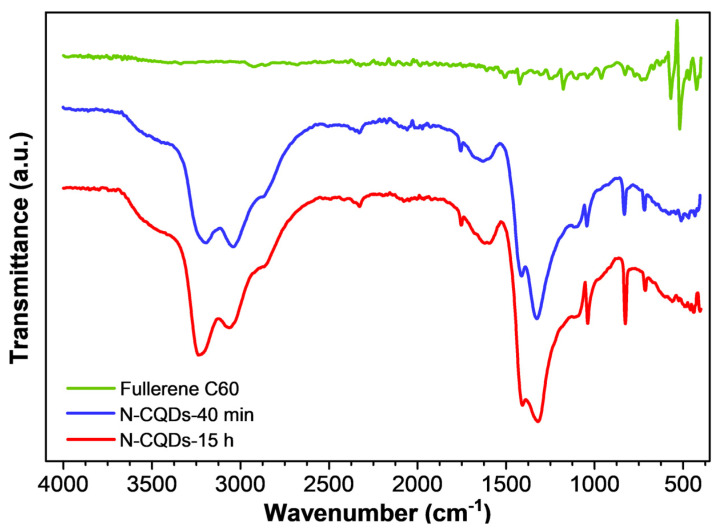
Infrared spectra of fullerene C60 (green solid line), N-CQDs-40 min (blue solid line), and N-CQDs-15 h (red solid line). The samples were deposited on clean Al foil via drop-casting prior to the measurement.

In Figure 3, the ATR-FT-IR spectrum of pristine fullerene powder is compared with the spectra of N-CQDs-40 min and N-CQDs-15 h.

Both N-CQDs samples appeared quite different with respect to the pristine fullerene powder since they showed broad absorptions in the region between 3500–2500 cm^−1^, as well as evident peaks below 1700 cm^−1^. In contrast, pristine fullerene was characterized by only four vibration modes located at 522 cm^−1^, 572 cm^−1^, 1180 cm^−1^, and 1427 cm^−1^ due to radial displacements of the carbon atoms (lower wavenumber peaks) and tangential modes of the carbon atoms (absorptions above 1000 cm^−1^) [43]. During the hydroxyl-radical-induced opening of fullerene, the surface of the obtained CQDs was modified by several functional groups, such as hydroxyl (–C-OH), carboxyl (-CHO, -COOH), ether and/or epoxy (-C-O-C-), amine (-C-NH_2_), and amide (-CO-NH_x_, x = 1, 2) moieties. The FT-IR spectra of the two samples of unfolded fullerene showed four broad absorptions between 3500 cm^−1^ and 2500 cm^−1^ due to the overlap of hydroxyl –OH stretching centered at 3440 cm^−1^ with -NH_x_ stretching (3199 cm^−1^), -CH=CH- (3044 cm^−1^) aromatic stretching, and -CH bond stretching of sp^3^ carbons (2860 cm^−1^). The most prominent peaks in this region were the –NH_x_ stretching and -C=C- aromatic stretching motions, indicating the formation of amine/amide group functionalized aromatic carbon particles. The weak but quite sharp peak registered at 1753 cm^−1^ could be assigned to –C=O carboxylic group stretching. The broad, weak signal centered around 1614 cm^−1^ conveyed signals from –OH bending, -C=C- aromatic stretching, -NH_2_ scissoring, and/or carboxylic acid -C=O stretching, thus hampering a one-to-one assignment [44]. The very intense and broad peak located at 1414 cm^−1^ could be related to –C-N- stretching of primary amides and to –C-O- stretching of hydroxyl groups in fullerenols [43,45]. Both N-CQD samples showed a prominent broad and intense absorption around 1319 cm^−1^, which could be ascribed to the –C-N- stretching vibration of secondary amines bound to fullerene moieties [43], as well as to the -C-O- stretching of the carboxylic acid bonds. The weak and broad absorption registered at 1093 cm^−1^ could be ascribed to the –C-O-C- ether group stretching and the weak and sharp signal at 1042 cm^−1^ derived from –C-C-O- asymmetric stretching of hydroxyl groups. Two sharp absorptions were observed in both samples below 1000 cm^−1^ and might have been related to the symmetric –C-C-O- hydroxyl stretching (827 cm^−1^) or –C-O-C- bending of isolated epoxy moieties on the CQDs’ surfaces [46]. Fingerprint wagging motions of –NH_2_ containing functional groups (amines, amides) were observed as a sharp absorption centered at 716 cm^−1^. After 15 h of reaction time, the intensities ascribed to -NH_x_ stretching (3236 cm^−1^) and –C-N- stretching of the amide groups (1408 cm^−1^) tended to increase. The largest increase of features occurred to the fingerprint -NH_2_ wagging, thus reasonably indicating a larger introduction of amine and/or primary amide groups over 15 h of synthesis. In addition, the peak assigned to epoxy group formation located at 827 cm^−1^ gained intensity in the N-CQDs-15 h sample, which might be ascribed to a more favorable/efficient cutting of the fullerene molecules. The summary of the IR bands of the samples and corresponding assignments is reported in Table 1. 

### 3.2. Optical Properties of N-CQDs-Based Nanomaterials

**Figure 4:**
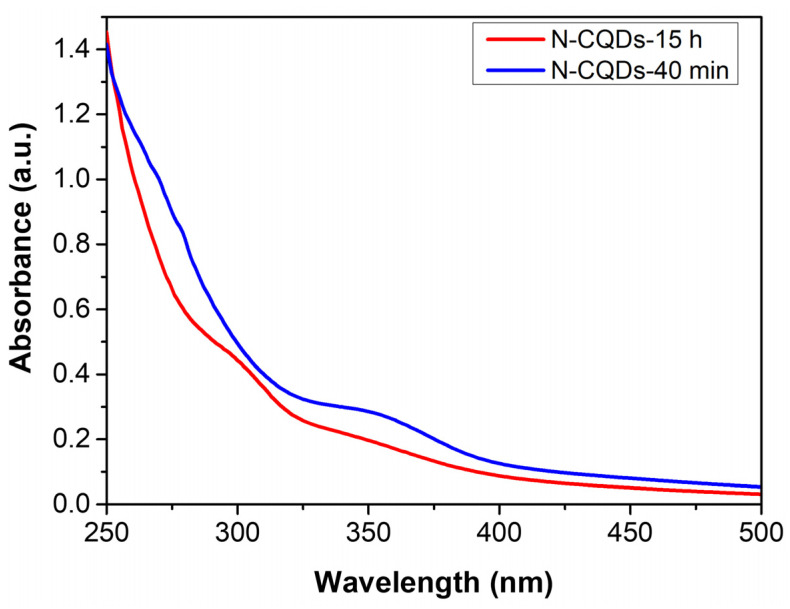
Absorption spectra of N-CQDs-40 min (blue solid line) and N-CQDs-15 h (red solid line).

The optical properties of the N-CQDs-40 min and N-CQDs-15 h were probed using UV-Vis and fluorescence spectroscopies. The prepared N-CQDs displayed slight differences in the absorption spectra. More in detail, the N-CQDs-40 min showed a typical absorption shoulder at 275 nm, whereas the N-CQDs-15 h presented a pronounced peak at 300 nm, which could be assigned to the π–π* transition of aromatic −C=C– bonds in the sp^2^-hybridized domain of the graphitic core (Figure 4). 

In addition, both samples showed a peak at 350 nm, which was more evident in the N-CQDs-40 min sample and could be assigned to the n–π* transition of −C=O, -C–N-, or −C–OH bonds in the sp^3^-hybridized domains. This transition could be related to hydroxyl (-OH), carboxylic (−COOH), or amine (−NH_2_) groups on the surface of CQDs [47,48,49,50]. 

**Figure 5:**
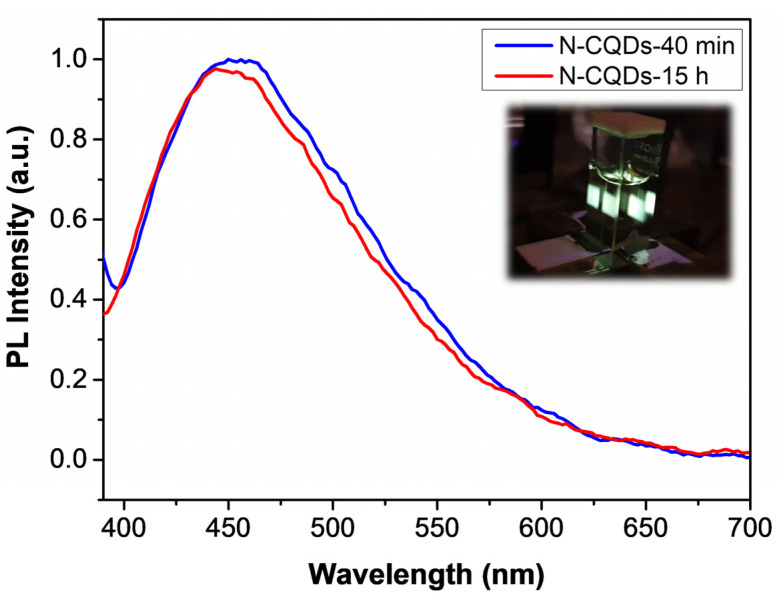
Normalized photoluminescent spectra of N-CQDs-40 min (blue solid line) and N-CQDs-15 h (red solid line). Inset: photograph of the N-CQDs solution excited at 360 nm.

In Figure 5, the PL spectra of the N-CQDs-40 min and N-CQDs-15 h excited at 360 nm are reported. Both samples displayed a blue-green emission in the visible spectrum with rather similar PL profiles. More in detail, fairly broad PL bands were observed with an FWHM of roughly 140 nm that peaked at 457 nm and 451 nm for N-CQDs-40 min and N-CQDs-15 h, respectively, highlighting a peak displacement that was compatible with the average size variation. The photograph in the inset of Figure 5 shows the emission of the N-CQDs upon excitation at 360 nm. 

The measured values of the fluorescence quantum efficiency of the two compounds were Φ = 4.5 ± 0.5% and Φ = 10 ± 1% for the syntheses over 40 min and 15 h, respectively. These values compared well with those found in carbon dots prepared through bottom-up strategies (though in the lower efficiency range). However, they were significantly higher than the value previously reported for undoped fullerene-derived quantum dots [51]. In this regard, the appreciable increase observed for 15 h of synthesis was consistent with the larger introduction of amine and/or primary amide groups indicated by the FT-IR spectra discussed above (see Figure 3) and agreed with the hypothesis that nitrogen introduces new surface states that trap electrons, thus easing radiative recombination [9,20]. The photostability of the samples was also good, with a fluorescence signal at 99.6% and 99.8% of the initial value for N-CQDs-40 min and N-CQDs-15 h, respectively, after 1 h of irradiation at 360 nm.

### 3.3. Selectivity of the N-CQDs as a Probe for Heavy Metal Detection

**Figure 6:**
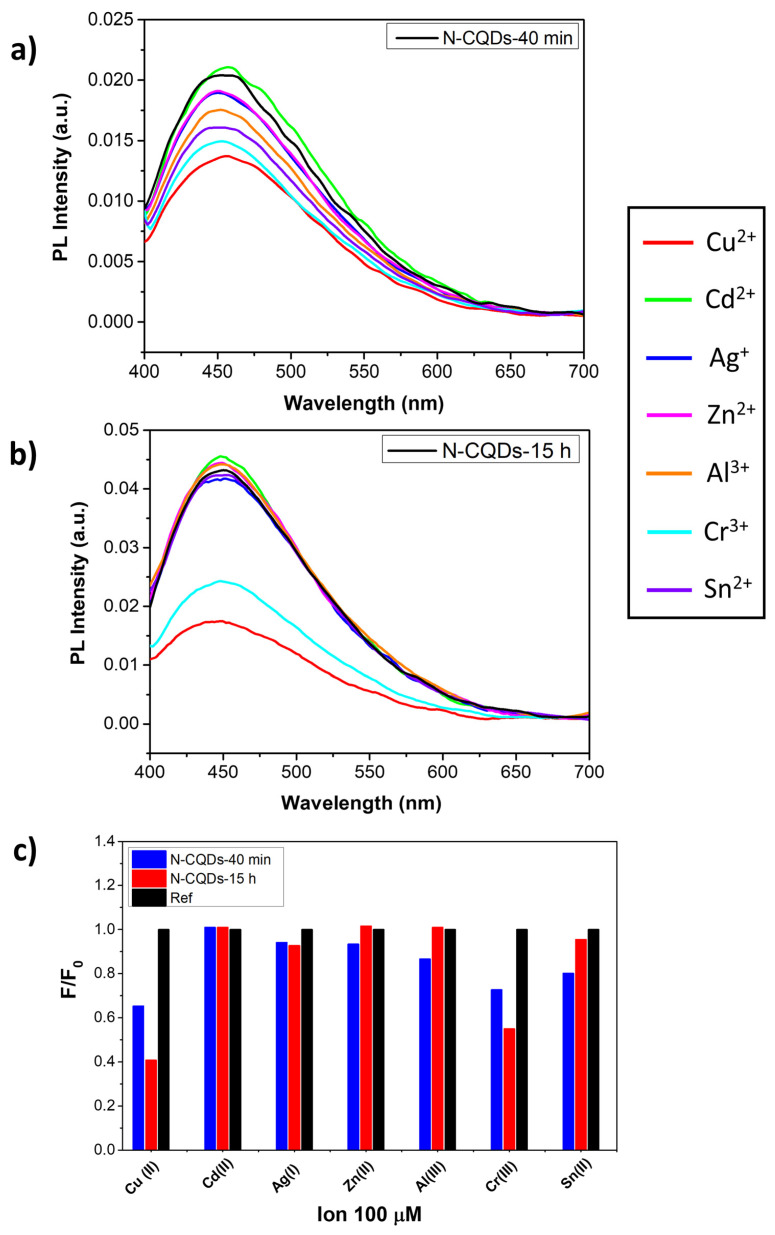
Photoluminescent spectra after excitation at 360 nm of (**a**) N-CQDs-40 min and (**b**) N-CQDs-15 h in the presence of different metal ions at the concentration of 100 µM. (**c**) Fluorescence quenching response of N-CQDs-40 min (blue bars) and N-CQDs-15 h (red bars) to different metal ions with the same concentration (100 µM). N-CQDs in the absence of metal ions are indicated as Ref. (black bars).

Selectivity is an important parameter to evaluate the performance of the N-CQDs as a fluorescent probe for heavy metal detection. Therefore, the fluorescence intensities of the N-CQDs were analyzed in the presence of various metal ions including Cu^2+^, Cd^2+^, Ag^+^, Zn^2+^, Al^3+^, Cr^3+^, and Sn^2+^ at the same concentration. Different studies [51,52,53] demonstrated that metal ions can interact with carbon quantum dots to induce quenching of the PL signal. In this scenario, the present study showed how the aqueous solutions of N-CQDs synthetized for different reaction times exhibited a significant response to two heavy metal ions: Cu^2+^ and Cr^3+^ but with different behaviors. In Figure 6a,b, the PL spectra of N-CQDs-40 min and N-CQDs-15 h upon interaction with the different metal ions are reported. N-CQDs-40 min displayed a fluorescence variation that depended on the metal in the solution. This fluorescence quenching was largest for Cr^3+^ and Cu^2+^ with intensity decreases of 25% and 32%, respectively (Figure 6a). At variance with this, N-CQDs-15 h showed comparatively very small fluorescence intensity variations with the various metals (Figure 6b), with the exception of Cr^3+^ and Cu^2+^, which caused significant quenching, both in comparison with the other metals and the interaction with N-CQDs-40 min. More in detail, the fluorescence intensity decreased by 44% and 60% for Cr^3+^ and Cu^2+^, respectively, whereas it was lower or negligible for the other ions. In summary, both the sensitivity and selectivity of N-CQDs-15 h were significantly higher as compared to N-CQDs-40 min. All these results are summarized in the histograms plot in Figure 6c, where F_0_ and F correspond to the fluorescence intensities of the N-CQDs at 452 nm in the absence and presence of metal ions, respectively. Assuming a linear behavior of the fluorescence quenching effect with decreasing ion concentrations, as is generally observed in fluorescent CQDs (see [28,29,30,31,50]), LODs of 2 μM and 1.5 μM could be estimated for Cr^3+^ and Cu^2+^, respectively. The LOD for Cr^3+^ just equals the current limit of 2 μM suggested by WHO for drinking water, whereas the LOD for Cu^2+^ is well below the current limit of 30 μM. 

The different behaviors of N-CQDs-40 min and N-CQDs-15 h with respect to Cr^3+^ and Cu^2+^ can be correlated with the larger presence of amine groups on N-CQDs-15 h, which may have given a more specific interaction, i.e., a larger affinity toward N-derivatives as compared to the other probed ions, such as in Reinecke’s salt (NH_4_[Cr(NCS)_4_(NH_3_)_2_]·H_2_O) or other stable Cr^3+^ complexes with N-derivatives, which can form with multiple bonds [54]. Similarly, Cu^2+^ may undergo a substitution of oxygen-dented ligands bonded to Cu^2+^ by nitrogen-dented ones [55], thus shifting the selectivity as well. 

The comparison with previously reported studies is limited because there are very few investigations on top-down syntheses of CQD for the detection of Cu^2+^ and we did not find any for the detection of Cr^3+^. Top-down syntheses of CQDs for Cu^2+^ detection were achieved using nitric or sulfonitric attacks of activated carbon or graphite fibers, respectively [56,57]. They both displayed a better LOD than N-CQDs-15 h, but the QY was not reported and no full assessment could be made. It must be added that the selectivity of the graphite-fiber-derived CQDs was achieved only via the addition of biothiol cysteine to the metal–CQDs water solution. A sulfonitric attack of carbon dusk in the presence of aminophenylboronic acid yields CQDs with a better LOD and worse QY as compared to our samples [58]. As for Cr^3+^, we made a comparison with bottom-up synthesized CQDs. In this case, we achieved both a better LOD and QY [59]. A summary of the data is reported in Table 2, along with the main synthesis details. 

### 3.4. Fluorescence of the N-CQDs—HEK293 Cell Cultures

**Figure 7:**
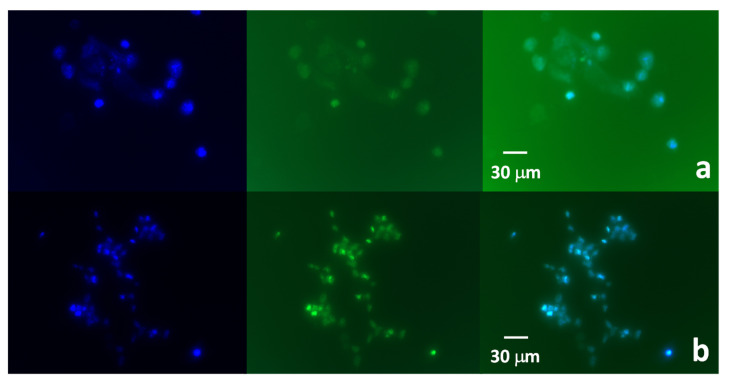
Images of the HEK-293 cells treated with (**a**) N-CQDs-40 min and (**b**) N-CQDs-15 h. Magnification: 40×. In each layer, the blue fluorescence of the nuclei stained with DAPI is reported, followed by the green fluorescence due to the N-CQDs and the overlap of the two.

The cultured HEK-293 cells, which were stained with DAPI and incubated for 30 min with either N-CQDs-40 min or N-CQDs-15 h, are shown in Figure 7a,b. In both cases, the HEK-293 cells could be clearly visualized after the internalization of the N-CQDs and exhibit a bright green fluorescence due to the particle endocytosis [60,61,62]. In addition, the fluorescence of N-CQDs-15 h was much more marked as compared to the N-CQDs-40 min. This could be attributed to a better piercing due to the smaller size of the N-CQDs-15 h, the -NH_2_ rich functionalization, and the higher quantum yield, which was twofold higher in the case of N-CQDs-15 h as compared to N-CQDs-40 min, or to a combination of the three factors. More importantly, the overlap of blue (nuclei) and green (N-CQDs related) fluorescence revealed the penetration of the N-CQDs in the nuclei membrane, although a slight diffusion in the cell cytosol was also visible. Typically, CQDs are cell markers of the cell cytoplasm [63,64]. 

Only recently, cases of nuclei penetration and marking were reported [65], such as the red-emission CQDs synthesized from hydrothermal treatment of p-phenylenediamine and doped with Ni, which showed the labeling of A549 cells nuclei, or the three-week-long electrochemical synthesis of CQDs from citric acid, followed by dialysis and used to stain the HEK-293 cell nuclei [66]. Comparatively, the present synthesis provided nuclei permeating N-CQDs without using potentially toxic material, such as Ni, and in relatively short time spans. The employment of N-CQDs-40 min or N-CQDs-15 h was also relatively safe since their incubation with HEK-293 for 1 or 2 h was characterized by cell viability of 99% and 98%, respectively, regardless of the type of quantum dots used.

## 4. Conclusions

In the present study, we successfully performed a one-step top-down synthesis of N-doped carbon quantum dots via hydroxyl-radical fullerene opening with H_2_O_2_ and NH_4_OH with different reaction times. The obtained N-CQDs were then characterized and probed regarding metal ion detection and bioimaging. We found a positive correlation between the reaction time, size, functionalization, and quantum yield. In particular, a longer reaction time (15 h) determined the formation, on average, of smaller quantum dots with a larger presence of -NH_2_ groups. This resulted in a metal ion selectivity shift toward the detection of Cr^3+^ and Cu^2+^ in an aqueous solution with a fluorescence intensity decrease by 44% and 60%, respectively, at 100 μM.

As far as the bioimaging properties are concerned, N-CQDs-15 h displayed a significant capability of penetrating and marking cell nuclei, which is a property that has been detected only for a limited number of CQDs so far. 

## Figures and Tables

**Table 1:** Main IR peaks of the pristine fullerene C60, N-CQDs-40 min, and N-CQDs-15 h, as well as the corresponding assignments; s = strong, m = medium, w = weak, vw = very weak, br = broad, sp = sharp, sh = shoulder.

Fullerene C60	N-CQDs-40 min	N-CQDs-15 h	Assignments
	3440 w br	3454 w br	-O-H stretching
	3199 m br	3236 s br	-N-H stretching
		3204 s sh	-N-H stretching
	3044 m br	3068 m br	-CH aromatic stretching
	2860 w br	2866 w br	-CH_x_ stretching
	1753 vw sp	1753 w sp	-C=O stretching
	1614 w br	1614 w br	-C=C-, -C=O stretching
			-NH_2_ scissoring
1427 m sp			-C=C- tangential displacement
	1414 w br	1408 s	-C-N-/-C-O- stretching
	1319 m–s br	1319 s br	-C-OH carboxylic acid stretching
	1093 w br	1101 w br	-C-O-C- ether stretching
1180 m sp			-C=C- tangential displacement
	1042 w sh	1041 m sp	-C-C-O- hydroxyl stretching
	827 m sp	827 s sp	-C-O-C- epoxy bending
	716 w sp	715 w sp	-NH_2_ wagging
572 s sp			-C-H radial displacement
522 s sp			-C-H radial displacement

**Table 2:** Comparison between N-CQDs-15 h and literature data of top-down synthesized CQDs for Cu^2+^ detection. For Cr^3+^, literature data of a bottom-up synthesis was taken. App = approach, TD = top-down, BU = bottom-up, QY = quantum yield, LOD = limit of detection.

Method	App	Precursors	Analyte	QY (%)	LOD (µM)	Ref.
Chemical oxidation	TD	Activated carbon was added to an HNO_3_ (5 mol/L) solution and refluxed at T = 125 °C for 72 h	Cu(II)	-	0.5	[56]
Chemical oxidation	TD	Graphite fibers in H_2_SO_4_ and HNO_3_ (3:1) were heated at T = 70 °C for 24 h	Cu(II)	-	0.33	[57]
Thermal decomposition	TD	Aminophenylboronic acid and carbon dusk in HNO_3_/H_2_SO_4_ were refluxed at T = 80 ℃ for 12 h	Cu(II)	1.6	0.3	[58]
Hydroxyl radical	TD	Fullerene in H_2_O_2_ and NH_4_OH were heated at T = 120 °C for 40 min or 15 h	Cu(II)	10	1.5	This study
Thermal treatment	BU	Sucrose and H_3_PO_4_ water solutions were incubated at T = 85 °C for 30 min	Cr(III)	0.18	24.5	[59]
Hydroxyl radical	TD	Fullerene in H_2_O_2_ and NH_4_OH were heated at T = 120 °C for 40 min or 15 h	Cr(III)	10	2	This study

## Data Availability

The data presented in this study are available on request from the corresponding author.
